# Supplementary figures and images for: A multiplex centrality metric for complex social networks: sex, social status, and family structure predict multiplex centrality in rhesus macaques
Source: PeerJ. 2020 Mar 16;8:e8712. doi: 10.7717/peerj.8712 (PMC7081788; doi:10.7717/peerj.8712)

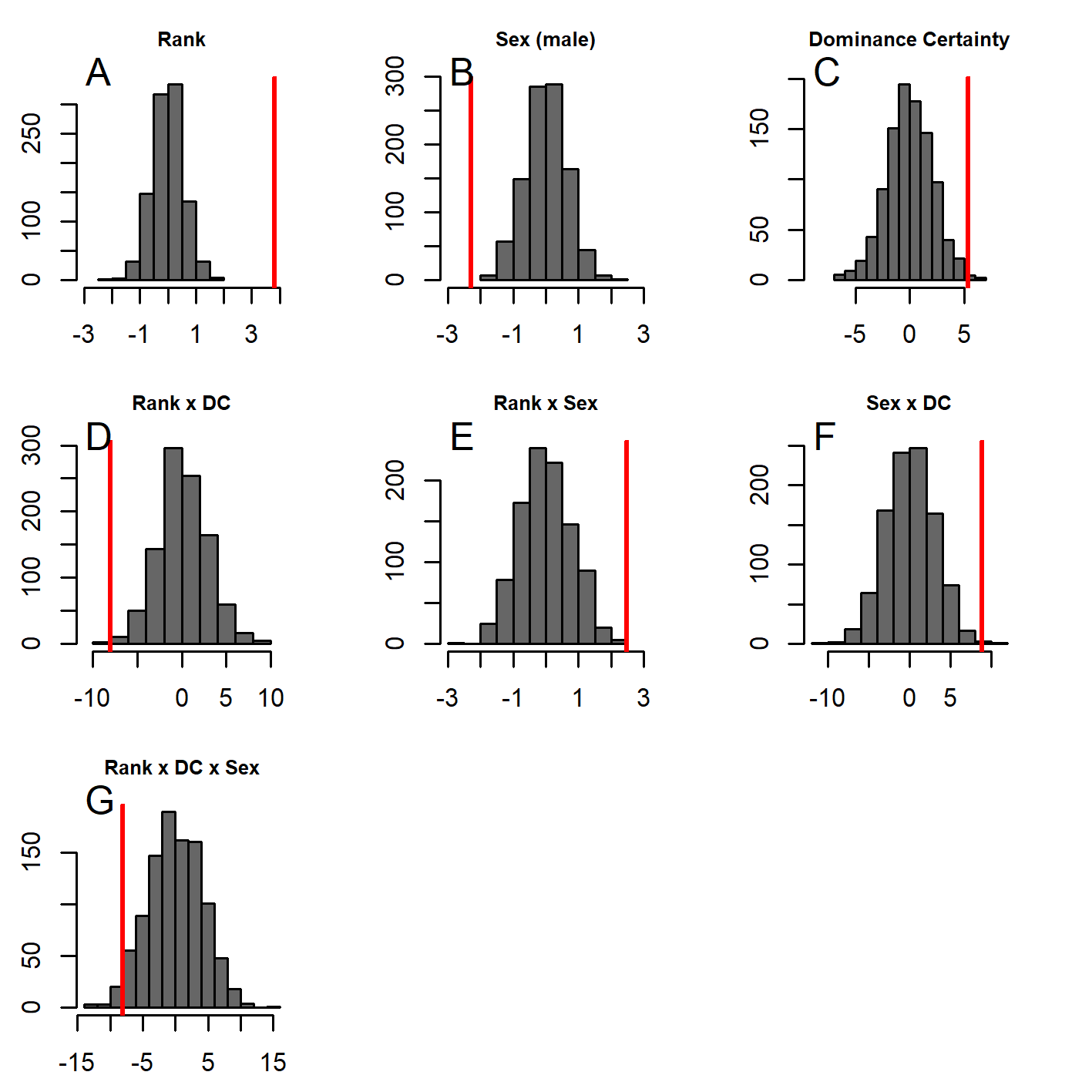

Supplement: Figure S1 — Histograms of coefficients for each predictor of centrality in the aggression layer, generated from fitting Model 1 to the network randomizations. For each randomization (n = 1000), all the node labels in the original network (e.g., sex, age, rank) were shuffled; then the same model (reversed aggression layer rank rank*sex*DC) was run for each randomized network. The vertical red line shows the value of the model coefficient for each predictor from the observed network data. [file peerj-08-8712-s002.png]

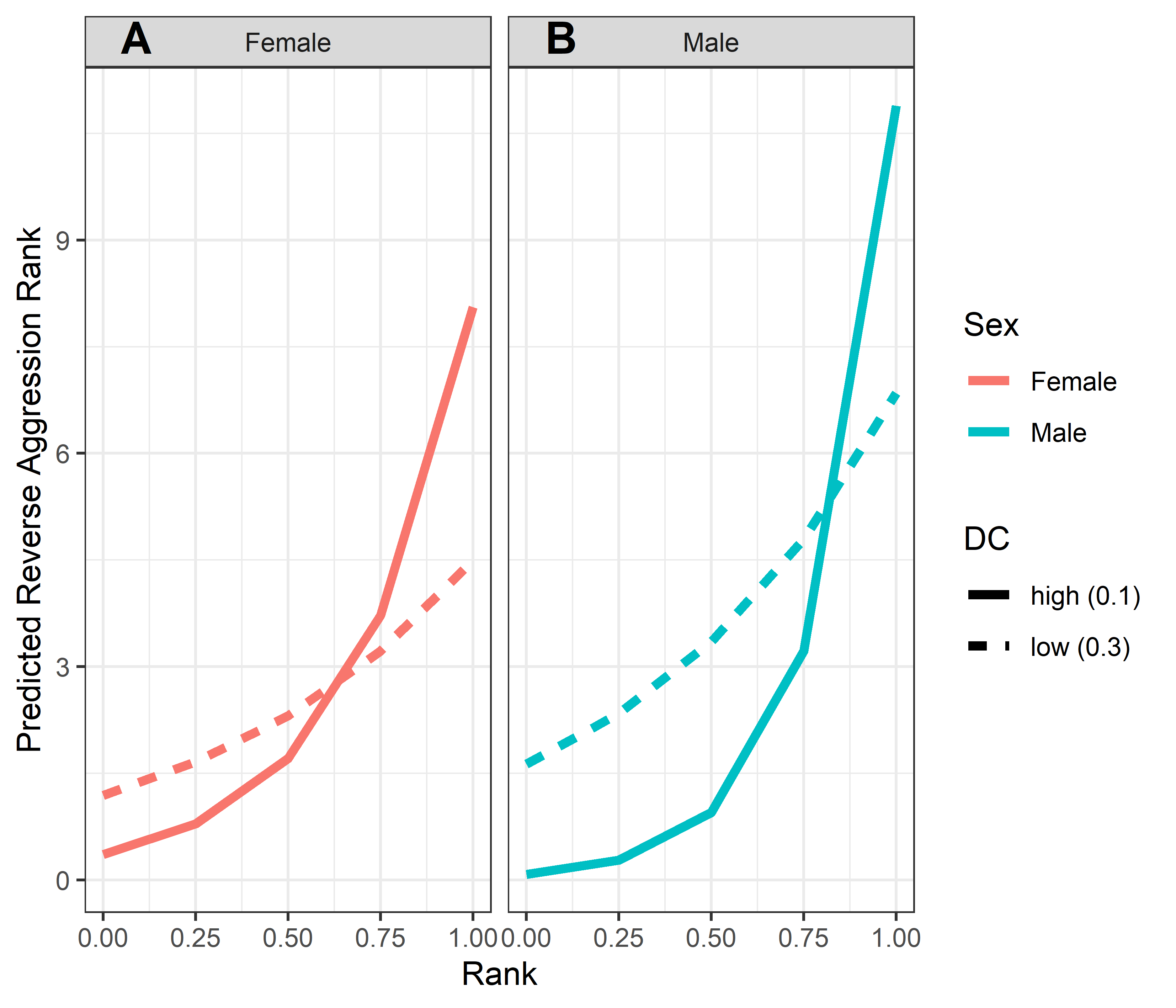

Supplement: Figure S2 — Predicted reverse aggression layer ranks for males and females, calculated at two levels of dominance certainty from the observed range of values: high dominance certainty (transformed DC = 0.1) and low dominance certainty (transformed DC = 0.3). Predicted values are based upon Model 1 of reverse aggression layer ranks. [file peerj-08-8712-s003.png]

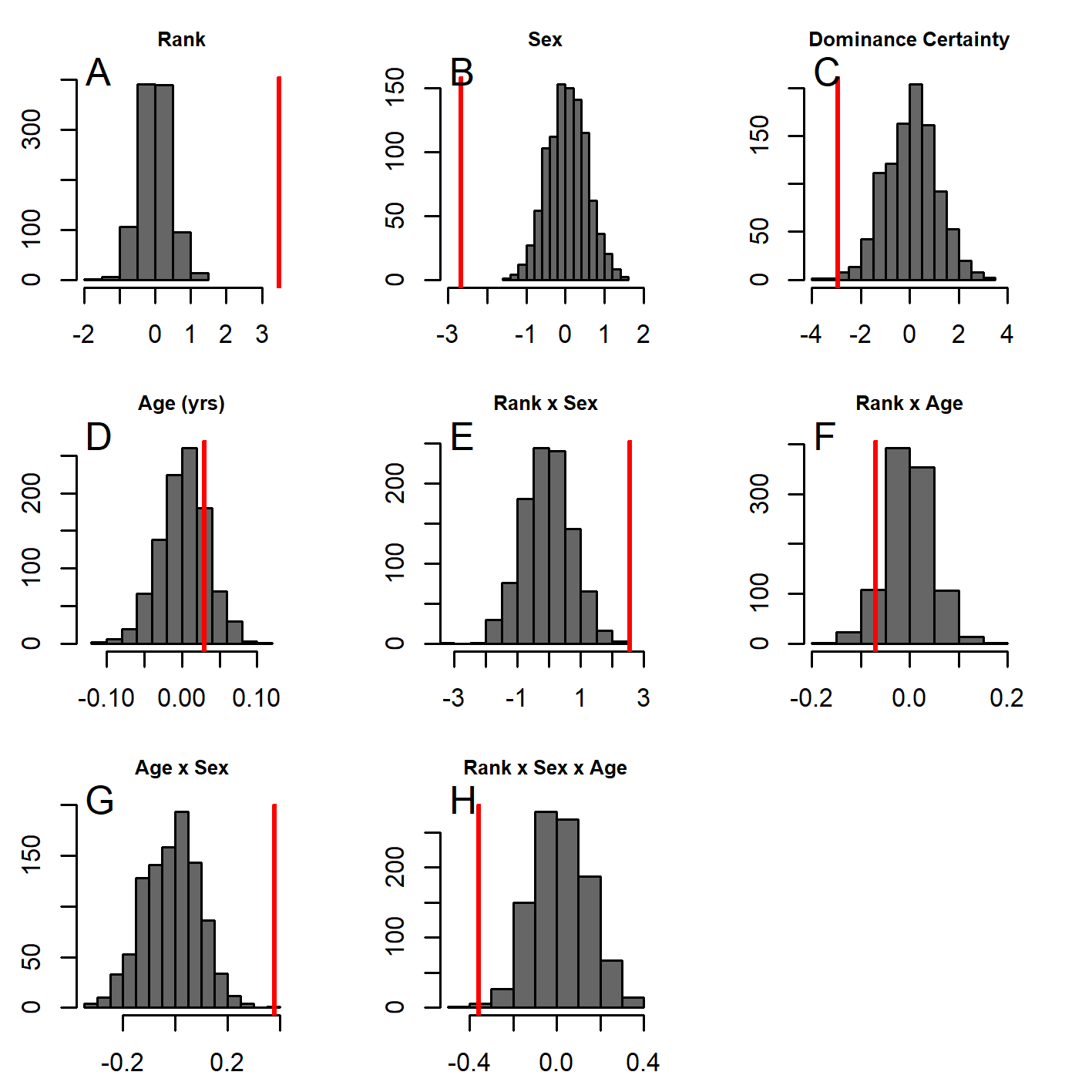

Supplement: Figure S3 — Histograms of coefficients for each predictor of centrality in the status signaling layer, generated from fitting Model 1 to the network randomizations. For each randomization (n = 1000), all the node labels in the original network (e.g., sex, age, rank) were shuffled; then the same model (reversed status layer rank rank*age*sex + DC) was run for each randomized network. The vertical red line shows the value of the model coefficient for each predictor from the observed network data. [file peerj-08-8712-s004.png]

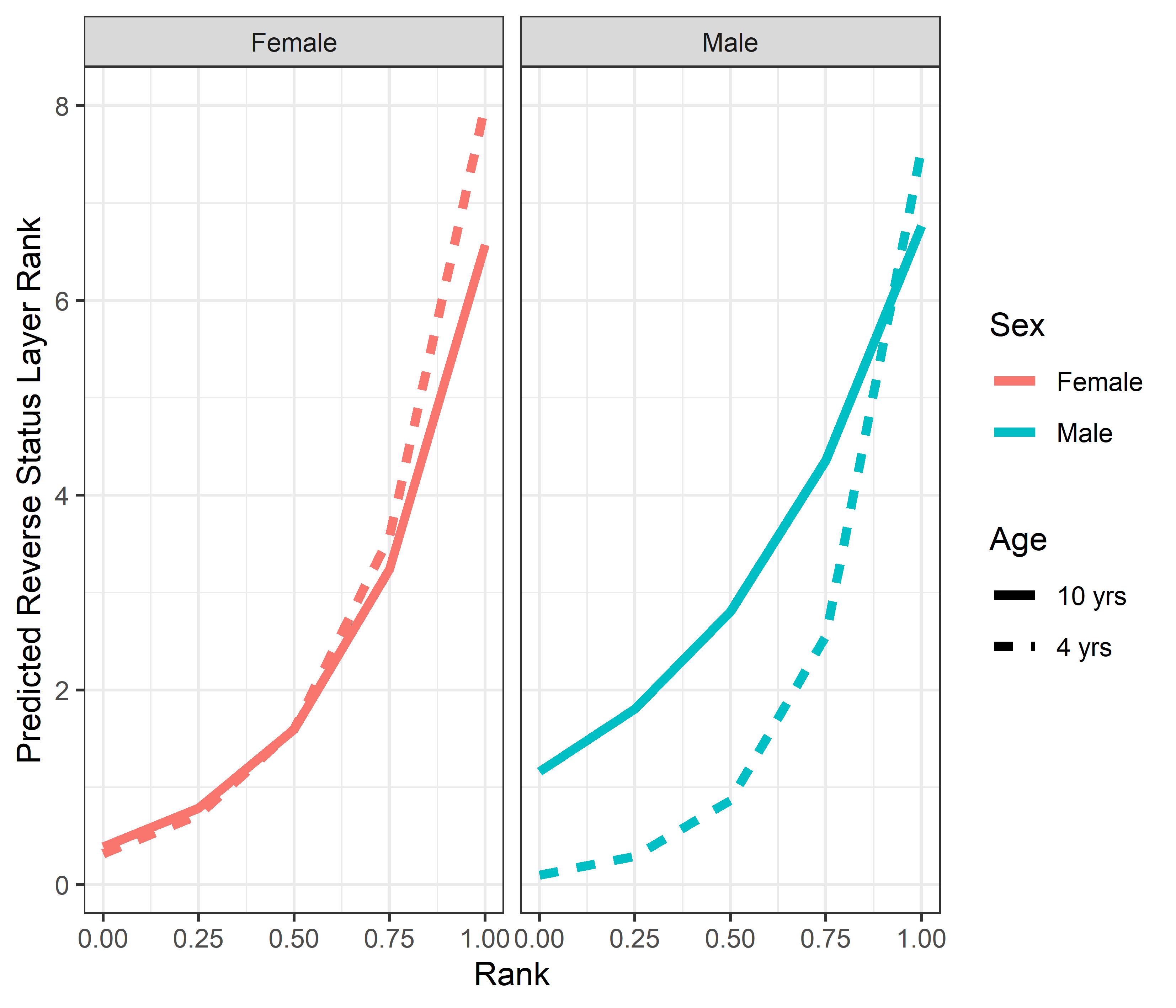

Supplement: Figure S4 — Predicted reversed status signaling layer ranks for females and males, calculated at two different ages (4 years and 10 years). Predicted values are based upon Model 1 of reverse status signaling layer ranks. [file peerj-08-8712-s005.png]

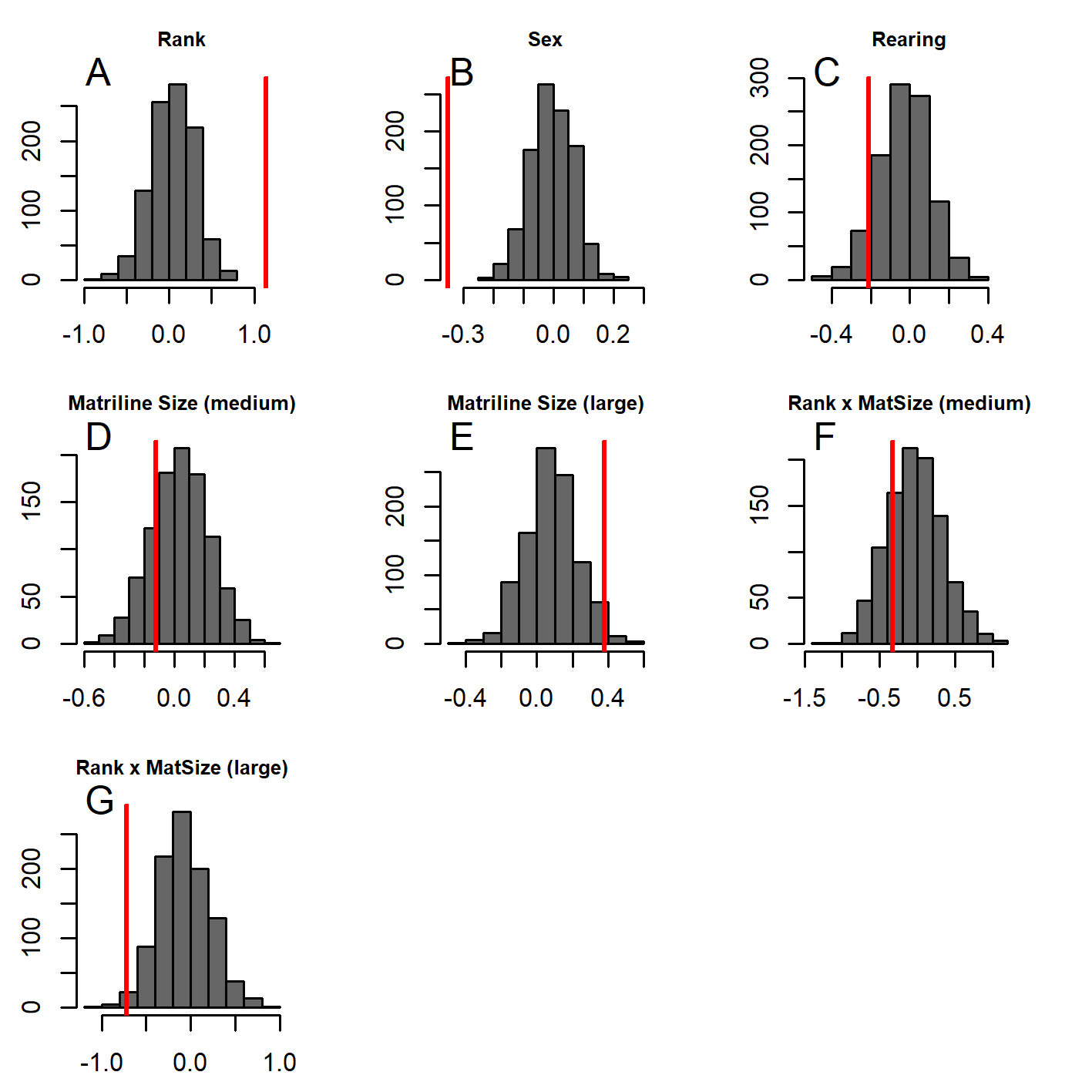

Supplement: Figure S5 — Histograms of coefficients for each predictor of centrality in the grooming layer, generated from fitting Model 1 to the network randomizations. For each randomization (n = 1000), all the node labels in the original network (e.g., sex, age, rank) were shuffled; then the same model (reversed grooming layer rank rearing history + sex + rank*matriline size category) was run for each randomized network. The vertical red line shows the value of the model coefficient for each predictor from the observed network data. [file peerj-08-8712-s006.png]
